# Is it possible to achieve multiplanar correction of complex deformities around the knee in children and adolescents using a monolateral external fixator?

**DOI:** 10.1007/s00264-024-06149-w

**Published:** 2024-04-01

**Authors:** Hassan Elbarbary, Ahmed Abdelmohsen, Abo-Bakr Zein, Amr Arafa, Mohamed Hegazy, Ahmed Yaseen, Ahmed Afifi

**Affiliations:** Department of Orthopaedic Surgery, Faculty of Medicine, Cairo University, Cairo, Egypt

**Keywords:** Monolateral external fixator, Deformities around the knee, Deformity correction in children and adolescents

## Abstract

**Purpose:**

To present the technique of correction of multiplanar deformities around the knee in children and adolescents using the monolateral external fixator. Also, to evaluate the results of the technique regarding radiological correction, time to union, and possible complications.

**Methods:**

A total of 29 patients (47 limbs) were prospectively included in the study (14 males and 15 females). Their median age was 13 years (range, 7–17). All patients had at least a 2-plane deformity around the knee which was corrected using a monolateral external fixator. The primary outcome measure was deformity correction (correction of mechanical axis deviation (MAD) in both the coronal and sagittal planes with correction of rotational deformities). The secondary outcome measures included bony union, radiographic, and functional results (assessed by using the Association for the Study and Application of the Method of Ilizarov (ASAMI) score).

**Results:**

The median pre-operative MAD improved from 6.3 to 0.4 cm post-operatively. According to the ASAMI scoring system, the radiographic scoring was excellent in all cases (100%), and the functional scoring was excellent in 22 cases (89.7%) and good in three cases (10.3%).

**Conclusion:**

The simple monolateral fixator can be an effective tool for multiplanar correction of complex deformities around the knee without limb length discrepancy.

## Introduction

Complex deformities around the knee are frequent during childhood and adolescence. These deformities distort the weight distribution on the affected joints leading to altered biomechanics of the limb with abnormal gait and disability [1, 2].

Proper deformity planning is the key to the management of limb deformity. A treatment strategy may occasionally aim at over or under correction of a deformity or even induction of a deformity in a normal bony segment to compensate for another [3].

Complex lower limb deformities are traditionally corrected via the Ilizarov external fixator with great success [4]. The frame potentially allows correction in any plane; however, in multiplanar deformities, it is sometimes challenging to position the hinges perfectly especially in combination with rotational deformities; thus, a staged procedure may be required to correct the deformity. Therefore, frame handling can be time-consuming, and changing the hinges may be necessary. Also, translation deformities may take place while correcting rotational deformities [4, 5].

The Taylor spatial frame (TSF) is used to permit simultaneous correction of multiplanar deformities with the use of a software program. Unfortunately, it is quite expensive and not always available [6–8].

Lengthening over nails has gained popularity for distraction osteogenesis in patients with lower limb deformities associated with limb length discrepancies. These nails proved to offer a better quality of life than external fixators during gradual correction of deformities in children [9].

The monolateral external fixator (MEF) has been used to correct angular deformities, especially coronal plane deformities (genu varum or valgum) [10]. Also, the use of MEF in complex deformity correction was introduced in the context of fixator-assisted nailing or plating in adults [11]. However, the literature is deficient in clinical trials that report the use of MEF alone for correction of multiplanar lower limb deformities.

This study aimed to present the surgical technique and the outcomes of correction of multiplanar deformities around the knee using the monolateral external fixator. The primary objective was correction of the mechanical axis with restoration of the planned joint orientation angles. The secondary objectives were time to radiological union, ease of use, and the rate of complications. The authors hypothesized that the monolateral fixator could achieve multiplanar correction if used properly.

## Materials and methods

### Design and setting

This single-center, prospective study was conducted at an academic level 1 paediatric orthopaedic center (Abo El-Reesh Paediatric University Hospital, Cairo University, Cairo, Egypt) between May 2020 and May 2023 after approval of the ethics committee.

### Participants

**Fig. 1:**
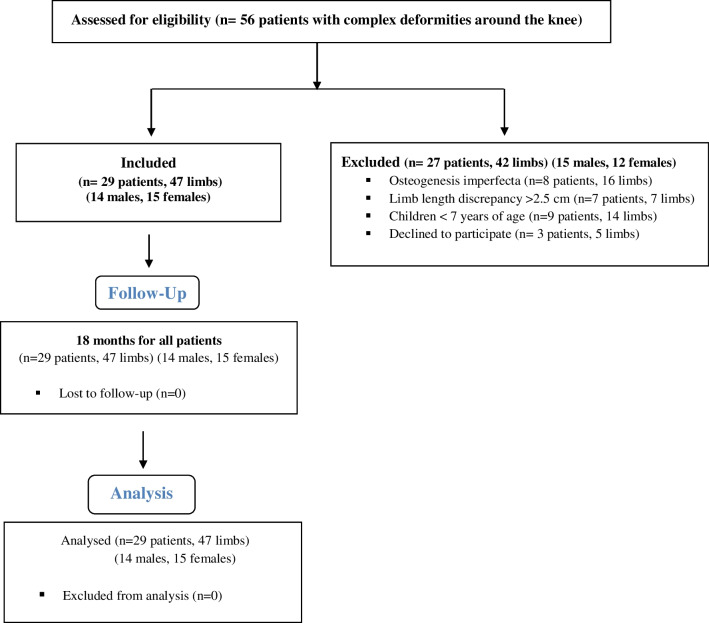
Flowchart of the participants

All patients with multiplanar deformities in the lower limb (coronal, sagittal, and/or rotational deformities) who presented to our outpatient clinic were registered and evaluated for eligibility (Fig. 1). The study included patients with at least two-plane deformities. We excluded patients with osteogenesis imperfecta, limb length inequality more than 2.5 cm, and children less than seven years of age (we prefer the cast-pin technique for them). Twenty-nine patients (15 patients with genu varum and internal rotation, and 14 patients with genu valgum and external rotation) (47 limbs) met the inclusion criteria and were enrolled in the study (Table 1). A written informed consent was signed by all parents/guardians of the participants included in the study after a detailed explanation of the treatment plan, the outcomes, and the possible complications. The study ended when all patients completed an 18-month follow-up period.

**Table 1:** Patients’ demographics

Age (median, min–max)	13, 7–17 years
Males:females (*n*, %)	14 (48.3%):15 (51.7%)
The affected side (*n*, %)	
○ Right	6 (20.7%)
○ Left	5 (17.2%)
○ Bilateral	18 (62.1%)
Deformity (*n*, %)	
○ Genu varum with internal rotation	15 (51.7%)
○ Genu valgum with external rotation	14 (48.3%)
Etiology (*n*, %)	
○ Rickets	16 (55.2%)
○ Post-traumatic	4 (13.8%)
○ Multiple hereditary exostosis (MHE)	5 (17.2%)
○ Blount’s disease	3 (10.3%)
○ Syndromic	1 (3.4%)

### Pre-operative evaluation

Standing scanogram radiographs (long films) were done for both lower limbs (anteroposterior and lateral views) with the knees fully extended and both patellae facing forwards. Computed tomography (CT) rotational profile was obtained to assess both femoral anteversion and tibial torsion. Serum calcium, phosphorus, and alkaline phosphatase (ALP) were ordered for all patients to screen for rickets and metabolic abnormalities. Optimization of the metabolic profile was done before surgery.

### Pre-operative planning

Deformity analysis was done using a software application (Bone Ninja version 5.0) created by the International Center for Limb Lengthening. X-ray scanograms were added to the application and processed to analyze both coronal and sagittal plane deformities.

### The monolateral fixator

**Fig. 2:**
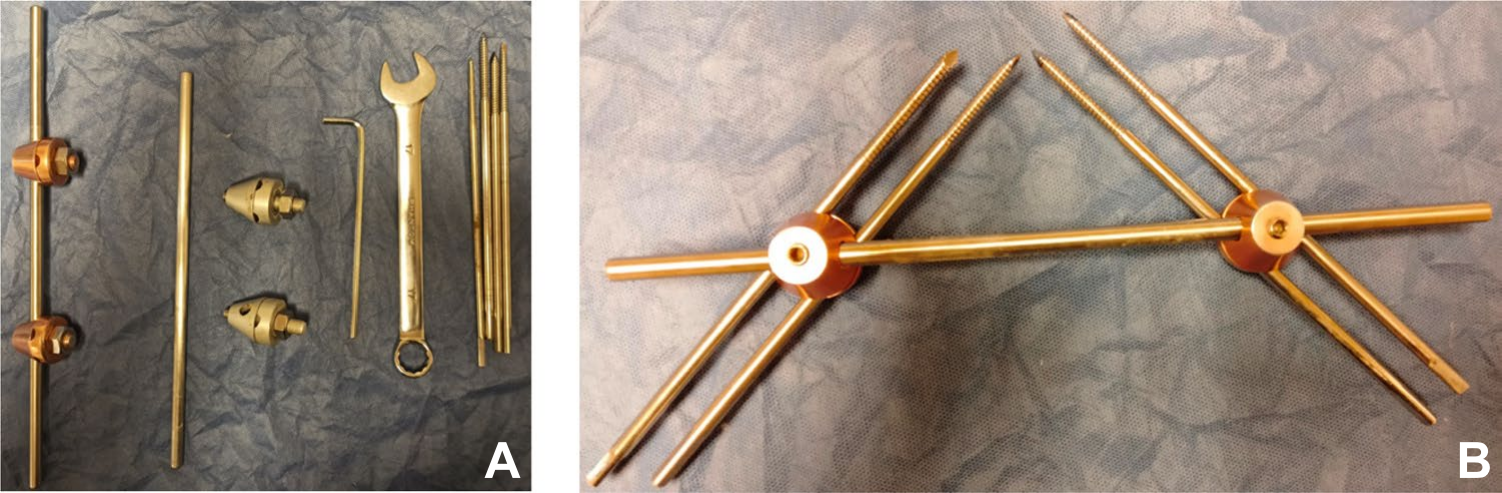
**A** The classic set used for application of the monolateral fixator. **B** Structure of the monolateral fixator

A locally made device was used. It was composed of pins (Schanz screws), clamps, and rods/bars. At least two proximal Schanz screws and two distal Schanz screws were connected via two clamps and one rod (Fig. 2). The clamps allowed the rotation of the Schanz screws which allowed the correction of large degrees of angular deformities.

### Plan of intervention

The idea was the correction of one deformity plane acutely and possible gradual correction/fine-tuning of the other deformity planes. In acute correction, we started with correction of rotation, then translation, then angulation. In gradual correction, the sagittal plane deformity was always fully corrected acutely while in the coronal plane deformity, Schanz screws were inserted parallel to the coronal plane so they could effectively correct any residual deformity.

### Surgical technique

#### Correction of axial rotational deformity

Proximal Schanz screws were inserted perpendicular to the bone segment (parallel to the ground) in the frontal plane. Distal Schanz screws were angled to the ground with the same angle of increased internal/external rotation determined pre-operatively. So, they were parallel to the position of distal segment deformity in the axial plane. The angle between the proximal and distal Schanz screws was the angle of the desired correction. A percutaneous transverse osteotomy was done between the two sets of Schanz screws. Correction was achieved via approximation of the proximal and distal Schanz screws to be parallel to each other.

#### Correction of sagittal plane deformity

**Fig. 3:**
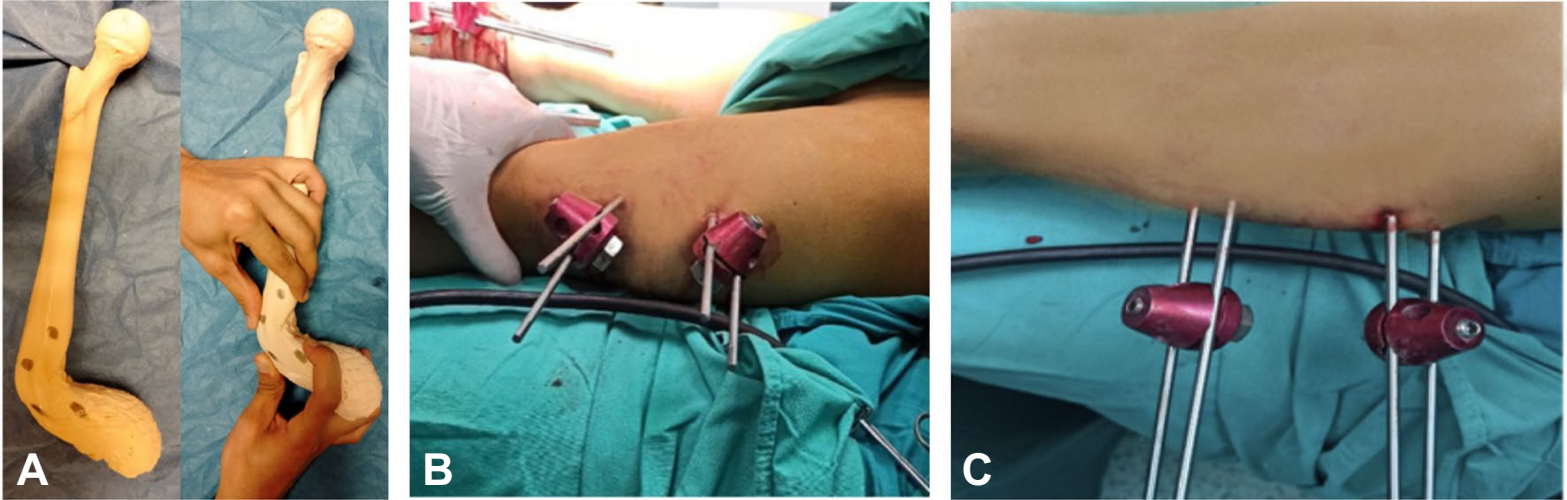
**A** A saw bone model showing the sites of insertion of Schanz screws according to thumb push technique. The sites of both thumbs and index fingers represented the positions of Schanz screws in the bone (black dots). This configuration allowed correction of sagittal plane deformity. **B**, **C** A case example showing the technique of correction of a sagittal plane deformity of the femur

The two proximal Schanz screws were inserted in the frontal plane; one Schanz screw was inserted relatively anterior in the circumference of bone relative to the other Schanz screw. The two distal Schanz screws were inserted in the same way as the proximal ones. A percutaneous transverse osteotomy was made between the two sets of Schanz screws with a large Schanz screw (5 or 6 mm) to produce crushing of a wedge of bone. The position of the clamps would be in an oblique manner. When the rod was inserted, clamps were realigned, and correction was achieved. Schanz screws were inserted according to the rule of thumb push; in cases of flexion deformity: near anterior, far posterior, parallel to the ground. In cases of extension deformity: near posterior, far anterior, parallel to the ground (Fig. 3).

#### Correction of coronal plane deformity

Two Schanz screws were inserted in the coronal plane parallel to the ground and parallel to the joint orientation line. The angle that was created between an imaginary mid-diaphyseal line and Schanz screws simulated the deformity present in the segment of the bone (epiphysis/metaphysis). Another two Schanz screws were inserted in the coronal plane perpendicular to the shaft of the bone segment. A transverse percutaneous osteotomy was made between the two sets of Schanz screws according to pre-operative planning. Corrections were made in the order of translation and then angulation via manipulation of Schanz screws. A rod over two clamps was used to maintain the correction. Gradual correction was possible via approximation or gradual separation of the Schanz screws in the follow-up period.

**Fig. 4:**
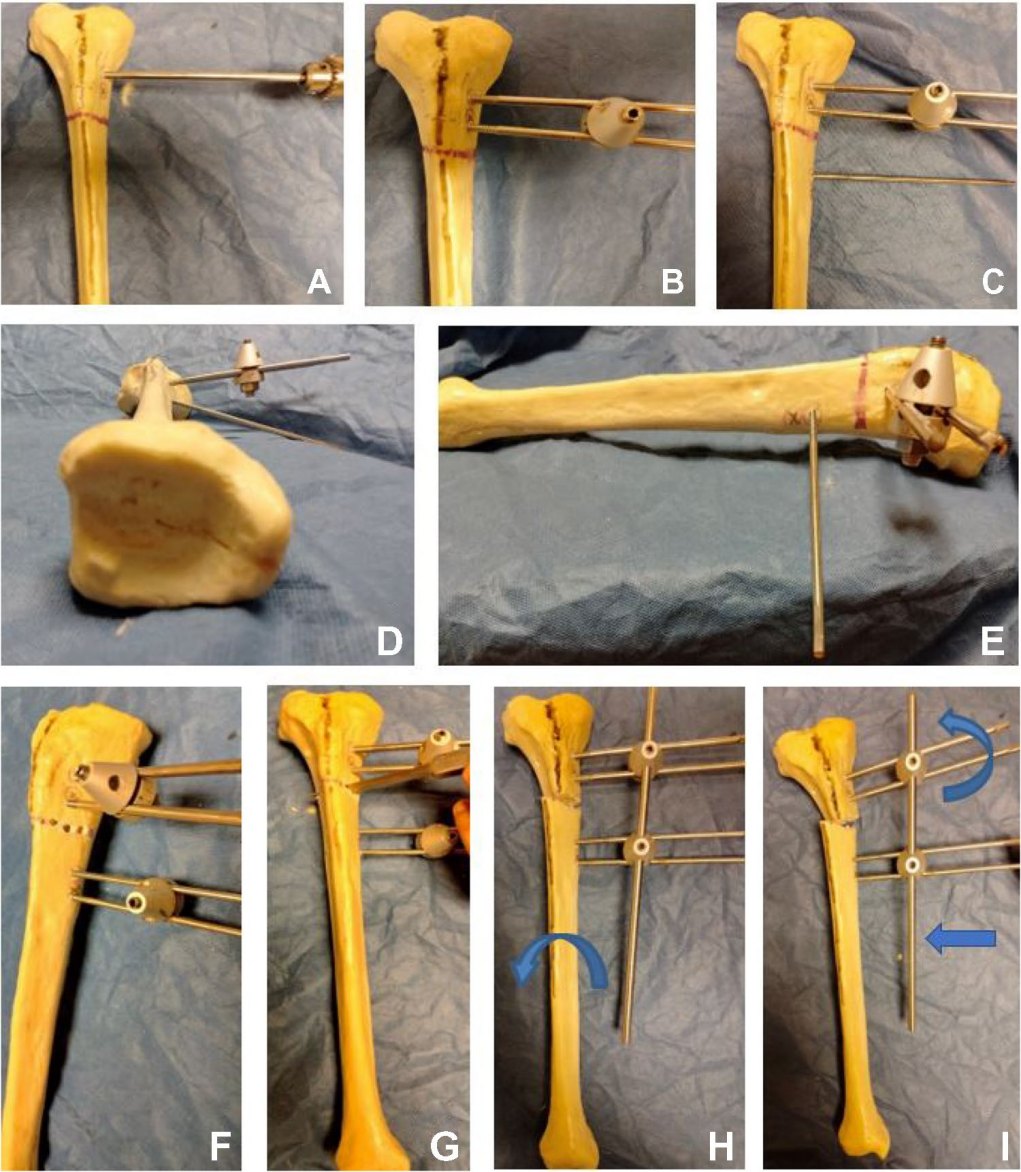
Demonstration of the technique of correction of tibial genu varum with internal tibial torsion using saw bones. **A**, **B** Insertion of the proximal Schanz screws parallel to the joint orientation line and parallel to the ground. **C**, **D**, **E**, **F** Insertion of the distal Schanz screws in the coronal plane and angled to the ground with same degree of the desired correction. **G** Transverse osteotomy was made in the planned site. **H**, **I** Corrections were made in the order of rotation then translation then angulation

**Fig. 5:**
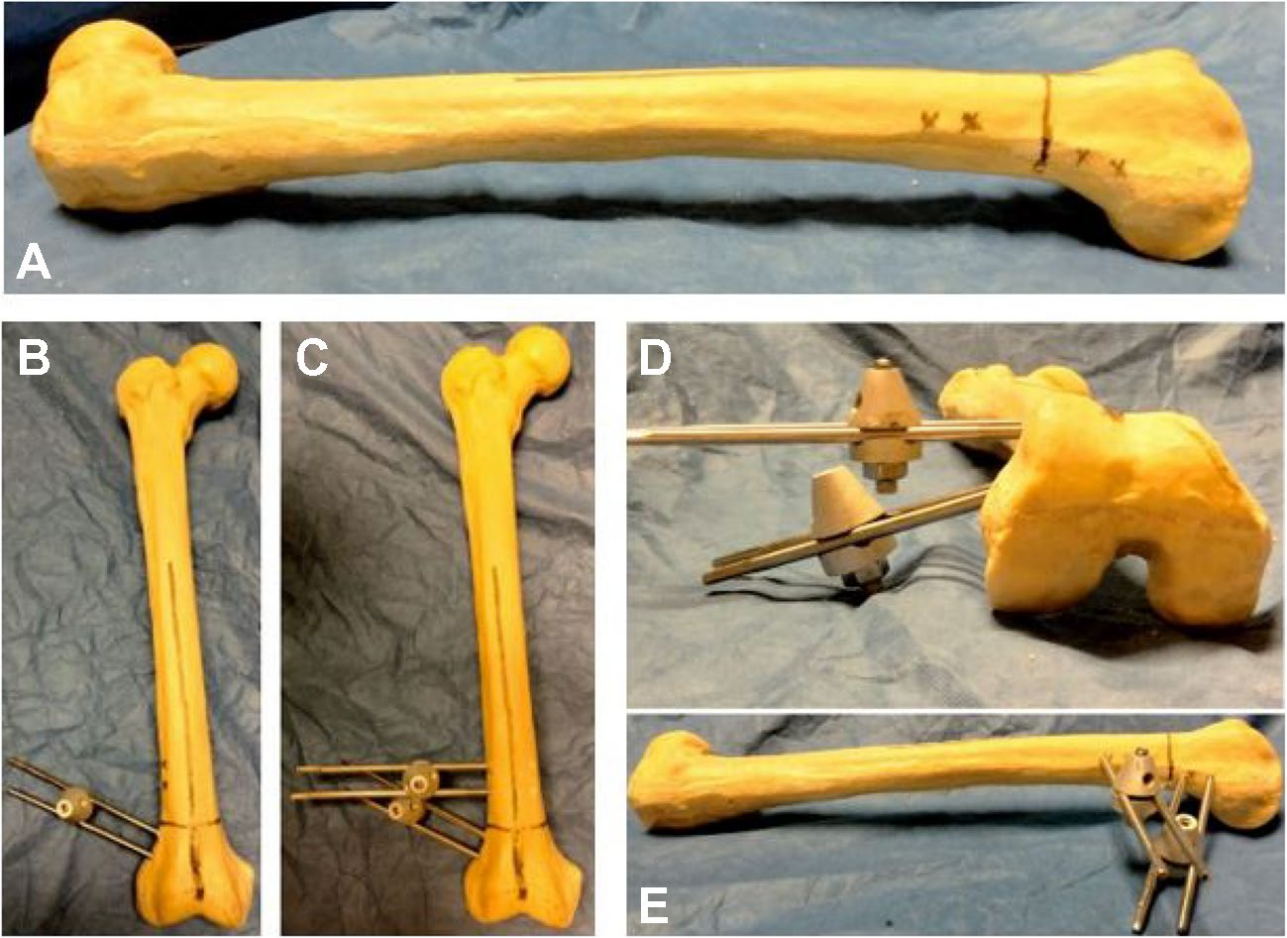
A saw bone model showing the technique of correction of femoral genu varum with external rotation. **A** The planned sites for insertion of distal and proximal Schanz screws along with planned osteotomy site. **B**, **C**, **D**, **E** Insertion of the distal Schanz screws, they should be angled to the ground with the same degree of external rotation

**Fig. 6:**
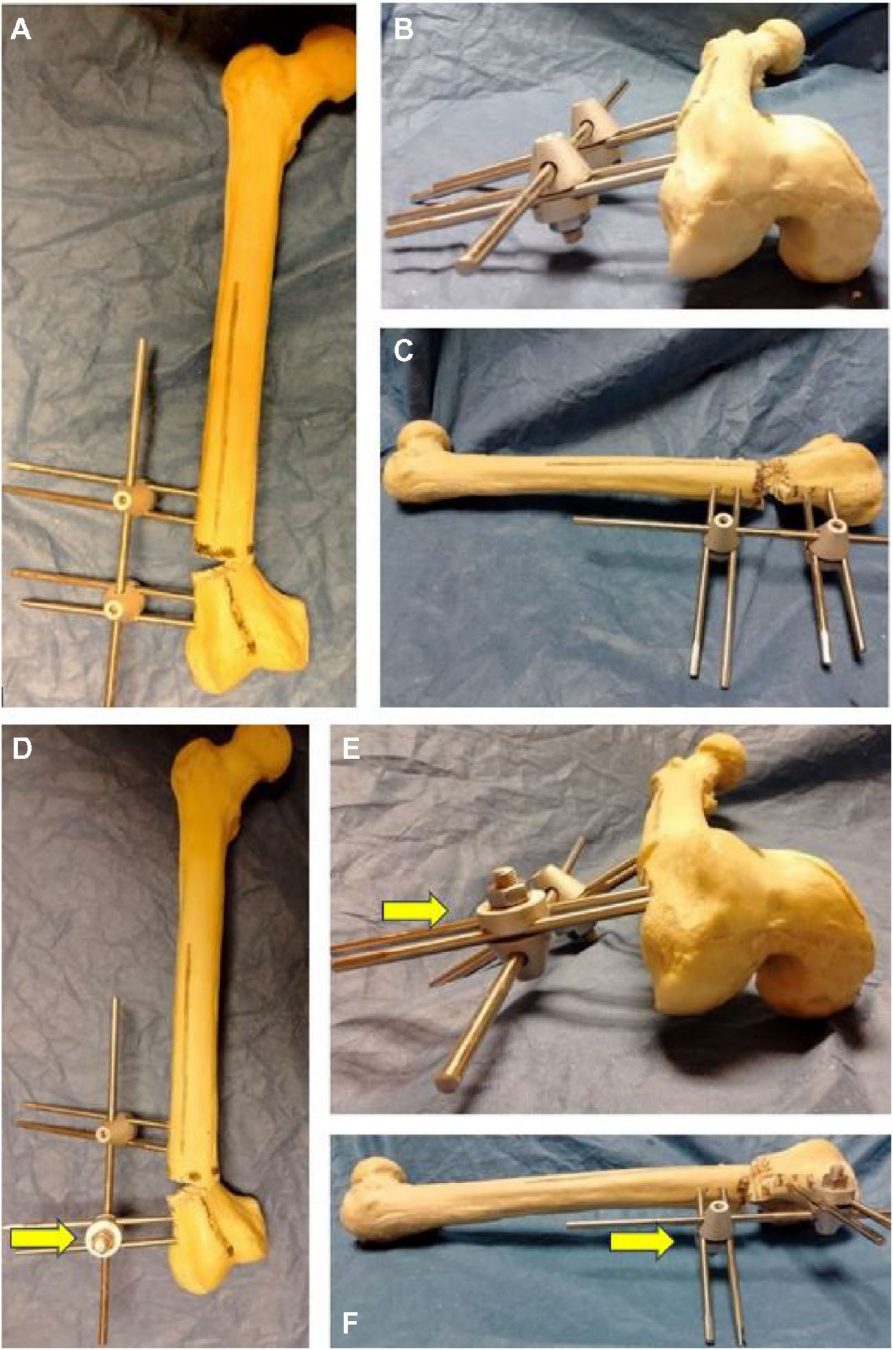
**A**, **B**, **C** Correction of external rotation deformity and genu valgum deformity. **D**, **E**, **F** Additional correction of rotation can be carried out via reversal of the distal clamp

Figure 4 shows a saw bone model for demonstration of the technique of tibial genu varum with internal tibial torsion. Figures 5 and 6 show a saw bone model for demonstration of the technique of femoral genu valgum with external rotation.

### Post-operative care

Patients stayed in the hospital till the next day to monitor any symptoms or signs of compartment syndrome. Neurovascular status was checked, e.g., dorsalis pedis and posterior tibial arteries pulsations and movements of the toes and ankle. Strict limb elevation was maintained on the operated side in the first week. The care of pin-insertion sites was taught to the mother or caregiver, e.g., cleaning with disinfectant solution (betadine) using a toothbrush with the removal of any crust or exudates.

Knee and ankle range of motion were encouraged from the first post-operative day. If the patients did not achieve adequate knee flexion (90°) by the end of first month, they were assigned to a physiotherapist. Weight-bearing was allowed after two to three weeks with the encouragement of the patients to do essential daily life activities, e.g., going to the bathroom.

### Follow-up program

Scanograms were done after two weeks to assess MAD, joint orientation angles, and residual deformities. Gradual correction was adjusted at two to three weeks post-operatively based on the pre-operative plan and post-operative scanograms. Follow-up radiographs were obtained at two, six and ten weeks, then every three weeks until fixator removal. Fixator removal was scheduled after confirmation of radiological union of three out of four cortices around the osteotomy site (in AP and lateral radiographs) and painless full weight bearing.

### Outcome measures

The primary outcome measure was deformity correction (correction of MAD in both the coronal and sagittal planes with correction of rotational deformities). Rotational correction was assessed clinically and by CT rotational profile. The secondary outcome measures included bony union, radiographic and functional results (assessed by using the Association for the Study and Application of the Method of Ilizarov (ASAMI) score) [12] (Table 2), and adverse events.

**Table 2:** Radiographic and functional scoring using ASAMI scoring system [11]

Bone results	
Excellent	Union, no infection, deformity < 7°, limb length discrepancy < 2.5 cm
Good	Union + any 2 of the following: absence of infection, < 7° deformity and limb length discrepancy < 2.5 cm
Fair	Union + only one of the following: absence of infection, < 7° deformity and limb length discrepancy < 2.5 cm
Poor	Non-union/re-fracture/union + infection + deformity > 7 + limb length discrepancy > 2.5 cm
Functional results	
Excellent	Active, no limp, minimum stiffness (loss of < 15 knee extension/ < 15 ankle dorsiflexion), no reflex sympathetic dystrophy (RDS), insignificant pain
Good	Active with 1 or 2 of the following: limp, stiffness, RSD, significant pain
Fair	Active with 3 or all of the following: limp, stiffness, RSD, significant pain
Poor	Inactive (unemployment or inability to return to daily activities of injury)
Failure	Amputation

Adverse events were collected according to Paley’s classification [13]; problems, obstacles, and true complications. Problems were post-operative difficulties that resolved completely with nonoperative intervention (i.e., superficial pin site infections). Obstacles were difficulties that needed operative intervention and resolved after surgery (i.e., contracture release). True complications were problems occurring intraoperatively and remained unresolved after treatment was completed.

### Statistics

Data were coded and entered using the Statistical Package for the Social Science (SPSS) software program version 21. Categorical variables were expressed as percentages. Numerical variables were expressed as a range, mean ± standard deviation if normally distributed, and median and interquartile range if not. Appropriate statistical tests of significance (the Wilcoxon test and correlation test for quantitative data) were used to test the null hypothesis in comparison between groups. The difference between groups was considered significant at *P*-value < 0.05.

## Results

The study included 47 limbs in 29 patients (14 males and 15 females). Their age ranged from seven to 17 years with a median of 13 years (Table 1). Fifteen patients had genu varum with internal rotation and 14 genu valgum with external rotation. The time needed for bony union after surgery ranged from two to four months with a median of 2.5 months.

**Fig. 7:**
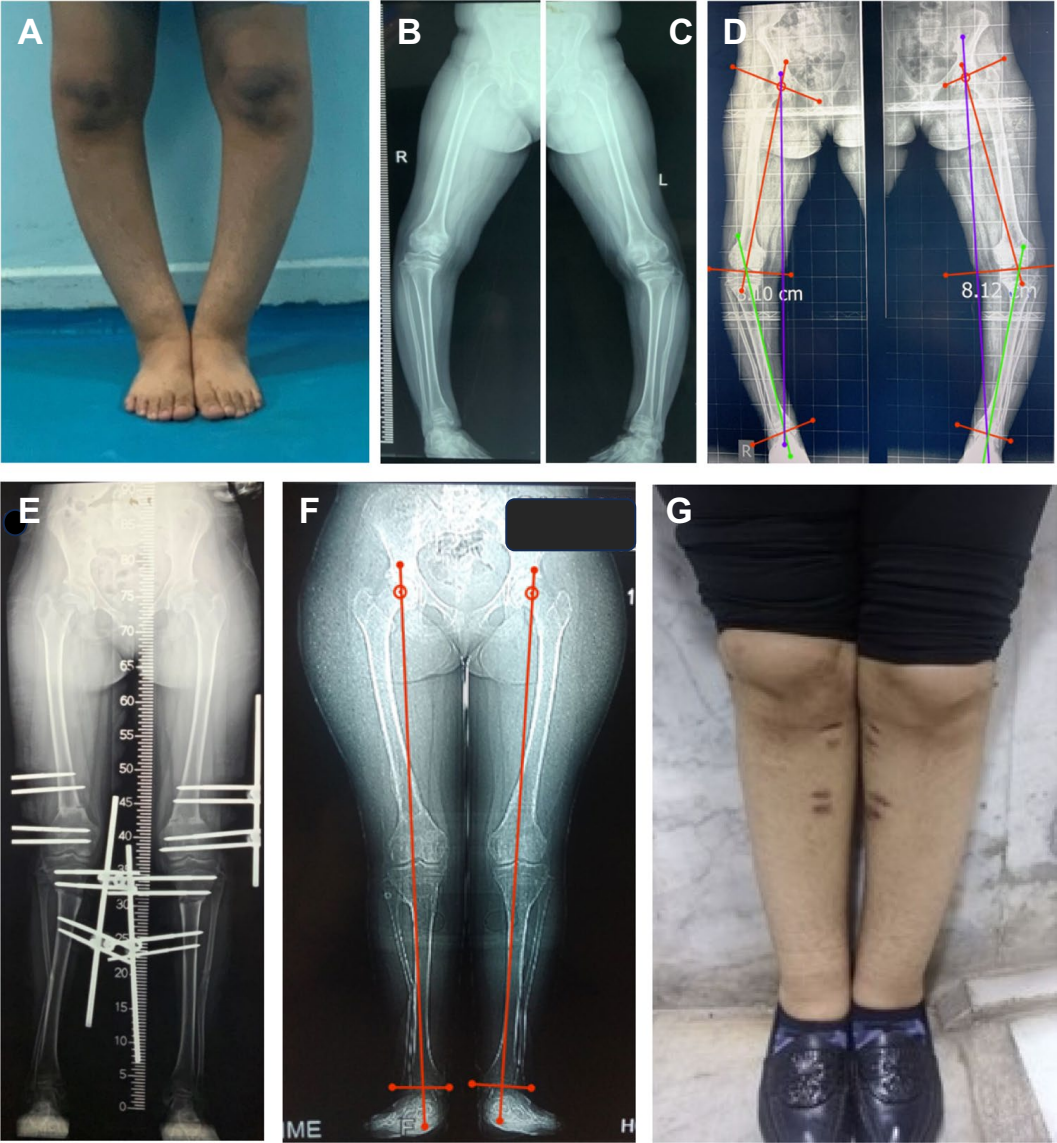
**A** A 14-year-old female patient with bilateral genu varum and internal tibial torsion. **B**, **C** Preoperative standing X-ray scanogram. **D** Preoperative measurement of joint orientation angles. **E** Postoperative scanogram after acute correction of the deformities using 2 monolateral frames. **F**, **G** Scanogram and clinical photo at the final follow-up after removal of the fixators

**Fig. 8:**
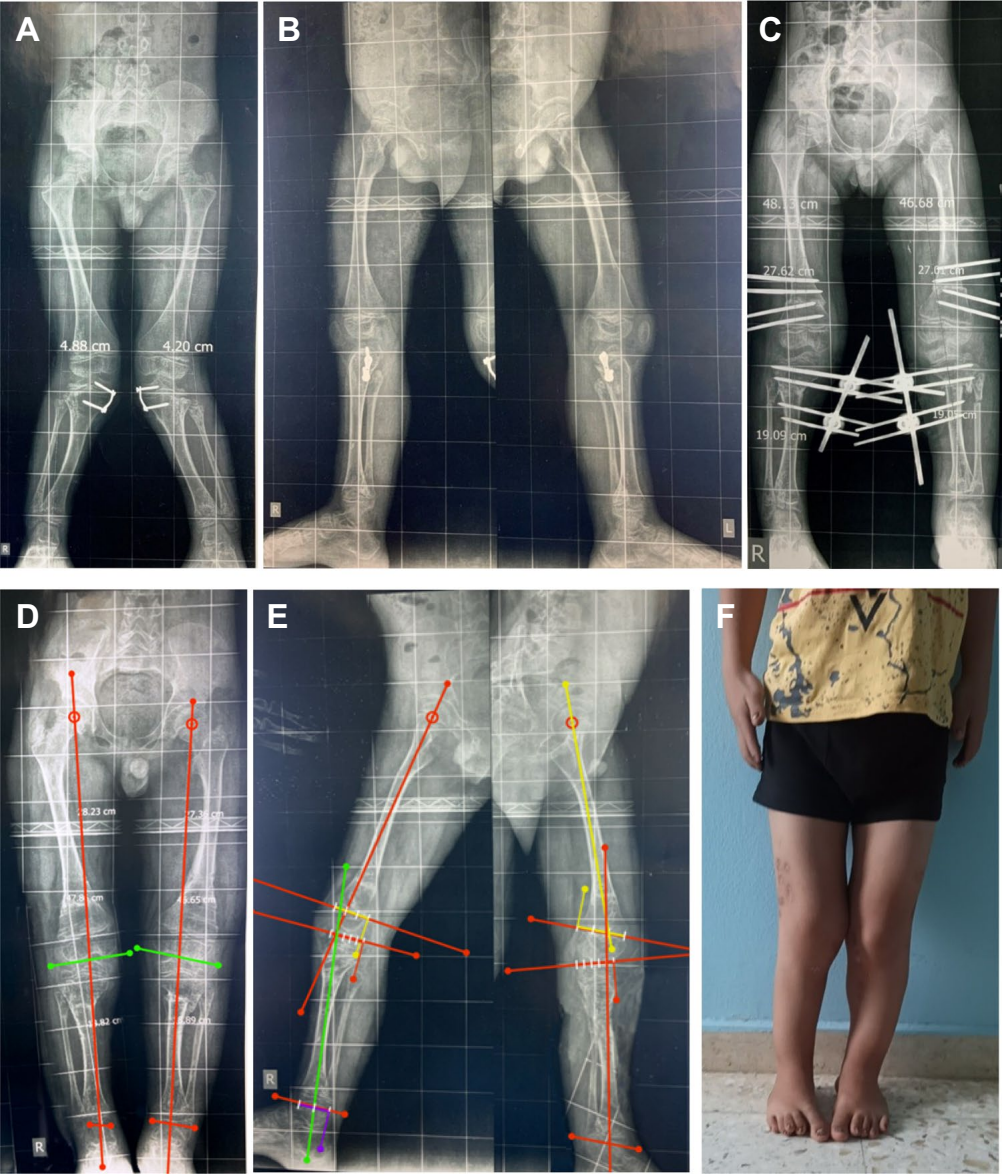
An 8-year-old boy with bilateral genu valgum and external femoral torsion (Ellis-van Creveld syndrome) after failed bilateral medial proximal tibial hemiepiphysiodesis. **A**, **B** Preoperative scanogram. **C** Postoperative scanogram after acute correction of the deformities using 2 monolateral frames for each limb. **D**, **E**, **F** Scanogram and clinical photo at the final follow-up after removal of the fixators

The pre-operative and post-operative angles are summarized in Tables 3, 4 and 5. According to the ASAMI scoring system, the radiographic scoring was excellent in all cases (100%), and the functional scoring was excellent in 26 cases (89.7%) and good in three cases (10.3%). Figures 7 and 8 show case examples with follow-up and clinical photos.

**Table 3:** Coronal plane correction in the whole sample (*n* = 47 limbs in 29 patients)

	Median	IQR	Min–max	*P*-value
Pre-op MAD (cm)	6.3	9–14	2–13	< 0.001
Post-op MAD (cm)	0.4	5–8	0.00–0.5	
Pre-op mLPFA	90.5	86.5–95	74–115	0.875
Post-op mLPFA	90	90–95	74–106	
Pre-op mLDFA	90.5	79–99	53–126	0.877
Post-op mLDFA	90	88.5–91	85–98	
Pre-op MPTA	88	77–100.5	72–117	1
Post-op MPTA	90	88–92.5	86–97	
Pre-op LDTA	85.5	80–88	68–106	0.041
Post-op LDTA	88	86.5–89.5	82–93	

*MAD mechanical axis deviation, *mLPFA* mechanical lateral proximal femoral angle, *mLDFA* mechanical lateral distal femoral angle, *MPTA* medial proximal tibial angle, *LDTA* lateral distal tibial angle

**Table 4:** Sagittal plane correction in the whole sample (*n* = 47 limbs in 29 patients)

	Median	IQR	Min–max	*P*-value
Pre-op SMAA	− 10	− 12 to 10	− 55 to 20	0.002
Post-op SMAA	0	0–6	0–12	
Pre-op SJLA	14.5	11–22	4 to 30	0.645
Post-op SJLA	15	12–20	0–34	
Pre-op PDFA	77	86–82	50–87	0.005
Post-op PDFA	82	80–83	71–124	
Pre-op PPTA	77.50	75.5–84	47–120	0.484
Post-op PPTA	80.00	79–82	70–88	
Pre-op ADTA	81.50	80–83	75–117	0.794
Post-op ADTA	82.00	81–85	79–90	

*SMAA sagittal mechanical axis angle, *SJLA* sagittal joint line angle, *PDFA* posterior distal femoral angle, *PPTA* posterior proximal tibial angle, *ADTA* anterior distal tibial angle

**Table 5:** Rotational “axial plane” correction in the whole sample (*n* = 47 limbs in 29 patients)

	Median	IQR	Min–max	*P*-value
Pre-op femoral version	20.00	7–40	5–50	0.155
Post-op femoral version	20.00	15–20	15–22	
Pre-op tibial torsion	40.00	20–45	15–50	0.003
Post-op tibial torsion	18.00	15–20	15–30

Complications of surgery are summarized in Table 6. The relapsed case was an eight year-old boy with vitamin D-dependent rickets. He had genu valgum with external rotation deformity. One year after correction, he had recurrence of the valgus deformity. He was scheduled for revision surgery after control of the metabolic profile.

**Table 6:** Complications of surgery

Complication type		Number of limbs	%
Problems	Superficial pin-tract infection	23	48.9
	Knee stiffness	4	8.5
Obstacles	Extensor hallucis longus (EHL) palsy	1	2.1
Complications	Relapse	1	2.1

## Discussion

In this study, the use of a simple monolateral external fixator achieved multiplanar correction with excellent and good outcomes in all cases and a low complication rate. Fewer complications were encountered than those reported in other studies which used TSF or Ilizarov. This could be explained by the longer duration of frame application due to distraction osteogenesis that was needed for limb lengthening and gradual deformity correction. This also could lead to the development of pin tract infection that might be deep-seated with possible subsequent osteomyelitis.

The workhorse of successful deformity correction is the percutaneous transverse osteotomy. It is a minimally invasive, low-energy osteotomy with minimal disruption of the periosteum and the surrounding soft tissues, e.g., muscles. This enhances mechanical stability and the biological environment for healing as soft tissues are minimally stripped. A transverse type of osteotomy can be easily performed via a percutaneous technique, and it allows easy translation as needed according to the pre-operative planning. Also, it allows deformity correction in more than one plane simultaneously, e.g., coronal and axial. This contrasts with the dome type of osteotomy that cannot correct the rotational element of the deformity.

The authors think that the art of deformity correction using a monolateral fixator depends on the technique and the direction of the insertion of the Schanz screws. These Schanz screws should be inserted to simulate the deformity in two planes at least. The stability of the monolateral frame is enhanced by adequate bone apposition at the osteotomy site despite the severe degree of deformity correction. This is due to the combination of translation with angulation to maximize the contact surface area. This occurs when the osteotomy level is away from the apex of the deformity, so obligatory translation is needed with angulation to achieve normal limb alignment.

The monolateral fixator is a well-known temporary fixation device in damage control orthopaedics (in polytrauma patients) [14]. Also, it can be utilized to correct angular deformities of the lower limb, especially coronal plane deformities (genu varum or genu valgum) [8]. The application of the monolateral fixator in deformity correction can be used as a step in the procedures of fixator-assisted nailing or plating; the monolateral fixator is used intraoperatively for planning and deformity correction then the plate or the nail is used to maintain the correction. The fixator is removed at the end of surgery [10, 15].

There is no consensus in the literature about the best management of complex paediatric lower limb deformities. Most of the reports are heterogeneous, including different aetiologies, different deformity analyses, and different lines of treatment. Usually, complex deformities are associated with limb length discrepancy, and the goals of treatment are different with no wide acceptance of corrections. Although the monolateral fixator has been widely used, the literature lacks studies that use it in the correction of multiplanar deformities. So, we compared our outcomes with the outcomes of monolateral fixator-assisted nailing or plating and with other hexapod external fixators (TSF) in previous studies.

Wahab et al. [15] retrospectively analyzed all patients who underwent deformity correction of the lower limb with fixator-assisted intramedullary nailing from 2010 to 2017. The study included 29 bones (16 femora and 13 tibiae) in 13 patients. Their mean age was 22.6 years (range, 14–46). Fixator-assisted nailing was done for them. The mean MAD of the lower limb improved from 3.9 cm pre-operatively to 1.8 cm post-operatively. The mean mLDFA improved from 78.2 ± 8.3° pre-operatively to 85 ± 5.2° post-operatively. The mean MPTA improved from 92.8 ± 8.3° pre-operatively to 89.9 ± 2.6° post-operatively. However, this study can be criticized for being retrospective with a small sample size.

Naqui et al. [16] and Riganti et al. [17] used circular frames to correct complex multiaxial deformities in the lower limb with good outcomes. The similarities between these studies and our study are the age group of the patients and the large degrees of correction. The differences are the acute correction in our study versus the gradual correction in theirs and the presence of limb length discrepancy in their studies which needed more time for union than our study that had no limb length discrepancies.

Rozbruch et al. [18] used fixator-assisted plating (FAP) in 36 lower extremities in 27 patients with a mean age of 33 years and a mean follow-up period of 16 months. Their hybrid technique combined the advantages of both internal and external fixation via minimal incisions. However, we believe that a major advantage of the monolateral fixator compared to internal fixation is the ability to correct residual deformities or fine-tune the accuracy of correction in the follow-up period.

Other advantages of the monolateral frame compared to circular frames or internal fixation include easier application, less operative time, minimal blood loss, and no need for open osteotomy that may compromise the periosteum and the soft tissue sleeve. Also, nails and plates are not suitable for acute correction of the metaphyseal deformities in the skeletally immature patients because there will not be enough fixation points near the physis in contrast to external fixation where pins can be inserted above or below the physis. However, the monolateral fixator may be not the ideal in multi-apical sagittal plane deformities in the femur as the Schanz screws should be inserted parallel to the major plane of correction, i.e., form anterior to posterior in the femoral shaft and this would transfix the quadriceps mechanism. Also, in severe deformities of the sagittal plane, there is a need for multiple wedge resections, and this requires extensive exposure with soft tissue releases. Those events will affect the stability and biology of the osteotomy site. Acute correction with intramedullary fixation or gradual correction with circular frames should be considered. Moreover, a major disadvantage of the monolateral fixator is that it cannot be applied in complex deformities with limb length discrepancies.

Our study is limited by the relatively small sample size, the short follow-up period, and the absence of a control group. Moreover, the study is limited by the heterogeneous nature of the included limb deformities which might affect interpretation of the outcomes. The authors recommend future studies that focus on one type of deformity. Table 7 summarizes the results of similar studies [4, 16, 17, 19, 20].

**Table 7:** Summary of similar studies

Study	Type	Deformity type	Number of patients	Average age	Procedure	Results	Outcomes		Complications
							ASAMI bone score	ASAMI function score	
Tetsworth and Paley [4]	Retrospective	Complex LL deformities	23 (28 limbs)	18.7 years	Ilizarov correction	-The mean MAD improved from 48 to 8.6 mm -The mean mTFA improved from 16° to 3°	–	–	36%
Manner et al. [18]	Retrospective, comparative	Complex LL deformities	155 (208 limbs)	13.2 years	Ilizarov was used in 79 cases, and the TSF was used in 129 cases	-Deformity correction was achieved in 90.7% in the TSF group, compared to 55.7% in the Ilizarov group - Higher percentages of excellent results in the TSF group	–	–	–
Naqui et al. [15]	Retrospective	Simple and complex LL deformities	53 (55 limbs)	10.7 years	TSF	An acceptable correction of deformity was achieved in 52 limbs	–	–	62.3%
Riganti et al. [16]	Retrospective	Angular LL deformities ± LLD	47 Group 1: 27 patients with LLD < 2 cm) Group 2: 20 patients with LLD ≥ 2 cm)	14.5 years	Truelok hexapod fixator system (TL-HEX)	–	Good to excellent in 100% of patients in group 1 and 85% in group 2	Good to excellent in 93% of patients in group 1 and 75% in group 2	7.4% (group 1) 10% (group 2)
Lim et al. [19]	Retrospective	Proximal tibial coronal plane deformity	8	Younger than 9 years	Monolateral external fixator with or without cross-pinning	88.3% of patients achieved post-operative MPTA within the normal range (85° to 90°)	–	–	No
Our study	Prospective	Complex deformities around the knee without LLD	29 (47 limbs)	13 years	Monolateral external fixator	–	Excellent in all cases (100%)	Excellent in 22 cases (89.7%) and good in 3 cases (10.3%)	61.7%

*LL lower limb, *LLD* limb length discrepancy, *TSF* Taylor spatial frame, *MAD* mechanical axis deviation, *mTFA* mechanical tibiofemoral angle, *MPTA* medial proximal tibial angle

In conclusion, the ease of application and adjustment of the monolateral fixator could make it an attractive cheap option for correction of complex multiplanar lower limb deformities in the absence of limb length discrepancies. The technique is easy to learn, reproducible, with a short healing period, and a low complication rate.

## Data Availability

Available.
